# Abundance of gut *Prevotella* at baseline and metabolic response to barley prebiotics

**DOI:** 10.1007/s00394-018-1788-9

**Published:** 2018-07-25

**Authors:** Jonna Sandberg, Petia Kovatcheva-Datchary, Inger Björck, Fredrik Bäckhed, Anne Nilsson

**Affiliations:** 1 Department of Food Technology, Engineering and Nutrition, Lund University, Lund, Sweden; 2 Food for Health Science Centre, Lund University, Lund, Sweden; 3 Wallenberg Laboratory, Department of Molecular and Clinical Medicine, University of Gothenburg, Gothenburg, Sweden; 4 Faculty of Health and Medical Sciences, Novo Nordisk Foundation Center for Basic Metabolic Research, University of Copenhagen, Copenhagen, Denmark

**Keywords:** *Prevotella*, *Bacteroides*, Barley, Prevention, Stratification, Glucose regulation

## Abstract

**Purpose:**

We previously showed that short-term intervention with barley kernel bread (BKB) improved glucose tolerance. However, glucose tolerance was not improved in a subset of individuals (non-responders) who were characterized by a low *Prevotella*/*Bacteroides* ratio. The purpose of the present study was to investigate if the baseline *Prevotella*/*Bacteroides* ratio can be used to stratify metabolic responders and non-responders to barley dietary fiber (DF).

**Methods:**

Fecal samples were collected from 99 healthy humans with BMI < 28 kg/m^2^ between 50 and 70 years old. The abundance of fecal *Prevotella* and *Bacteroides* was quantified with 16S rRNA quantitative PCR. 33 subjects were grouped in three groups: subjects with highest *Prevotella*/*Bacteroides* ratios, “HP”, *n* = 12; subjects with lowest *Prevotella*/*Bacteroides* ratios, “LP”, *n* = 13; and subjects with high abundance of both measured bacteria, HPB, *n* = 8. A 3-day randomized crossover intervention with BKB and white wheat bread (control) was performed. Cardiometabolic test variables were analyzed the next day following a standardized breakfast.

**Results:**

The BKB intervention lowered the blood glucose responses to the breakfast independently of *Prevotella*/*Bacteroides* ratios (*P* < 0.01). However, independently of intervention, the HP group displayed an overall lower insulin response and lower IL-6 concentrations compared with the LP group (*P* < 0.05). Furthermore, the groups HP and HPB showed lower hunger sensations compared to the LP group (*P* < 0.05).

**Conclusions:**

Here we show that the abundance of gut *Prevotella* and *Bacteroides* at baseline did not stratify metabolic responders and non-responders to barley DF intervention. However, our results indicate the importance of gut microbiota in host metabolic regulation, further suggesting that higher *Prevotella*/*Bacteroides* ratio may be favorable.

**ClinicalTrials.gov ID:**

NCT02427555

**Electronic supplementary material:**

The online version of this article (10.1007/s00394-018-1788-9) contains supplementary material, which is available to authorized users.

## Introduction

Lifestyle habits play a pivotal role for the development of obesity, type 2 diabetes (T2D) and cardiovascular disease (CVD) [1]. Diet is probably the most significant lifestyle factor for the etiology of metabolic diseases, making the dietary composition an essential target in a strategy aiming at prevention [1]. Recent studies have demonstrated that saturated fats and animal protein, which are typically high in a Western diet, are associated with metabolic disorders such as obesity, reduced insulin sensitivity, T2D, and CVD [2], and also altered microbiota, e.g., reduced diversity [3]. In contrast, inclusion of whole grain foods in the diet has been found advantageous by lowering the risk of weight gain, T2D, and CVD [4]. Accumulating evidence from both epidemiological studies [5] and dietary interventions [6–9] suggest preventive potential of dietary fiber (DF) on cardiometabolic risk. The beneficial effects of DF are largely suggested to emanate from mechanisms related to microbial metabolism of the DF [6–10]. We and others have shown that barley kernel-based evening meals improve glucose tolerance following a subsequent standardized breakfast [6, 7, 10]. The underlying mechanisms were suggested to emanate from colonic fermentation of the DF present in barley. Recent studies have observed a high variability in the effects on glucose tolerance to food between subjects although the meals or more specific food items were identical [11, 12]. Thus, the importance of personalized nutrition is highlighted and it has been suggested that the glucose response can be predicted by collecting data regarding personal features and/or gut microbiota composition [11, 12]. In agreement, we previously reported high variability in glucose response in test subjects following barley kernel-based evening meal [10]. Interestingly, we found that the microbiota differed between the subjects acquiring improved glucose tolerance after intake of barley (responder subjects) compared to subjects that showed no or minor effects on glucose tolerance (non-responder subjects). In responder subjects, intake of barley was associated with increased abundance of gut *Prevotella* and increased ratio of *Prevotella*/*Bacteroides*. In contrast, in non-responders, the abundance of *Prevotella* or ratio *Prevotella*/*Bacteroides* was not affected after barley kernel-based products. In addition, we observed tendencies towards an increased ratio of *Prevotella*/*Bacteroides* in the responder subjects compared with the non-responder subjects already at baseline, i.e., prior to the barley intervention [10]. The purpose of this study was to investigate if the ratio *Prevotella*/*Bacteroides* at baseline can be used to identify and stratify metabolic responders from non-responders to barley DF in a population.

## Materials and methods

### Test subjects

Recruitments of subjects were conducted through advertisements in local newspapers during May and June 2015. The screening was conducted at the Food for Health Science Centre, Lund University. The inclusion criteria were as follows: men and women aged 50–70 years with normal to slightly increased body mass index (BMI < 28 kg/m^2^), normal blood glucose concentration as determined with a random blood glucose test < 8.7 mmol/l, overall healthy and with unknown metabolic disorders or food allergies. Subjects with anti-hypertensive medications (3 subjects) and prescription-free painkillers without anti-inflammatory action were allowed to participate. In addition, no antibiotics or probiotics should have been consumed within 3 weeks prior to fecal donation, during the selection process and throughout the following dietary intervention. In total, 99 apparently healthy volunteers (as judged from the inclusion criteria), 25 men and 74 women, aged 64.1 (SD 5.6) years, with a BMI of 24.3 kg/m^2^ (SD 3.4 kg/m^2^) and with a random blood glucose concentration of 5.5 mmol/l (SD 0.8 mmol/l), donated stool samples at baseline, i.e., prior to start of the dietary intervention.

The study was conducted in accordance to the Declaration of Helsinki, and all procedures involving human subjects were approved by the Regional Ethical Review Board in Lund, Sweden (Reference 2015/58). Written informed consent was obtained from all the subjects. The study was registered at ClinicalTrials.gov (NCT02427555).

### Genomic DNA purification and 16S rRNA quantitative PCR analysis

Fecal samples were collected at baseline from all 99 subjects. The sample was immediately frozen (− 20 °C) and handed over to the experimental department within 24 h for continued storage at − 80 °C until analysis. Genomic DNA was purified from the feces of 99 subjects, using repeated bead beating [13]. 16S rRNA quantitative PCR was performed with a CFX96 Real-Time System (Bio-Rad Laboratories). Samples were analyzed in a 25-µl reaction mix consisting of 12.5 µl 1xSYBR Green Master Mix buffer (Thermo Scientific, Waltham, Massachusetts, USA), 0.2 µM of each primer and 5 µl of template DNA, water or genomic DNA. All reactions were performed in duplicate in one run. qPCR conditions were as reported previously: for *Bacteroides* and universal [14]; for *Prevotella* [15]; Standard curves of 16S rRNA PCR product of *B. thetaiotaomicron, P. copri* or *Escherichia coli* (for universal 16S rRNA qPCR) were created using serial tenfold dilution of the purified PCR product.

### Selection of individuals for the short-term dietary intervention

The selection of the individuals for the intervention study with barley kernel bread (BKB) and white wheat bread (WWB) was based on their baseline microbiota composition. Main criteria for the subgrouping were the abundance (high ‘H’ or low ‘L’) of *Prevotella* and *Bacteroides* and the ratio of *Prevotella*/*Bacteroides* in their baseline gut microbiota. Individuals with highest levels of *Prevotella* and low levels of *Bacteroides* and ratio of *Prevotella*/*Bacteroides* more than 1.0 were identified as high *Prevotella* (HP) subjects. Individuals with low levels of *Prevotella* and high levels of *Bacteroides* and the ratio of *Prevotella*/*Bacteroides* equal to 0 were identified as low *Prevotella* (LP) subjects. Out of the total cohort of 99 subjects, we identified 15 HP subjects. Based on this number, we selected 15 LP subjects with the lowest *Prevotella* levels and the highest Bacteroides levels. Some of the individuals (*n* = 10) had high levels of *Prevotella* and high levels of *Bacteroides* and were identified as high *Prevotella* and *Bacteroides* (HPB) subjects and also included in the intervention study.

An additional inclusion criterion for participation in the short-term intervention study was that the fasting level of plasma glucose should be below 6.1 mmol/l, i.e., below the level of impaired fasting glucose (6.1–6.9 mmol/l) according to the World Health Organization definition [16]. Six participants were excluded due to fasting plasma glucose > 6.4 mmol/l (LP, *n* = 2; HP, *n* = 1 and HPB, *n* = 1) or a BMI > 30 kg/m^2^ (HP, *n* = 1 and HPB, *n* = 1). Due to the exclusions, the statistical evaluations were based on 12 subjects in the HP group, 13 subjects in the LP group and 8 subjects in the group HPB.

### Test (BKB)—reference (WWB) products

The test product BKB consisted of a barley kernel-based bread containing 85% barley kernels (Finax) and 15% white wheat flour, presented as % cereal dry matter (dm). The reference product WWB consisted of a white wheat-based bread and was based on 100% (dm) white wheat flour (Kungsörnen AB). The WWB was also included in the standardized breakfast. The BKB and WWB were characterized with respect to total starch [17], resistant starch (RS) [18], and insoluble and soluble non-starch polysaccharides (NSP) [19]. Further details regarding characterization using these methods are provided elsewhere [6]. The starch and NSP compositions are presented in Table 1.

**Table 1:** Characterization of test and reference product with respect to starch (total, available and resistant) and NSP

Starch					NSP		Total DF
Products	Daily amounts	Total	Available^a^	RS	Insoluble	Soluble	
% dry matter							
BKB	–	65.5	57.2	8.4	8.4	4.0	20.8
WWB	–	76.1	74.1	2.0	3.9	2.0	7.9
g/day							
BKB	384	115	100	14.7	14.7	7.0	36.4
WWB	259	103	100	2.7	5.3	2.7	10.7

Data are presented as means

*BKB* barley kernel bread, *RS* resistant starch, *NSP* non-starch polysaccharides, *DF* dietary fiber, *WWB* white wheat bread

^a^Available starch was obtained by calculating the difference between total starch and RS. Values of total and available starch are based on means of 2 replications, RS means of 6 replications, DF means of 3 replications

The ingredients and baking procedure of BKB and WWB are in accordance with previously used procedures [6], except that the barley kernels were boiled for 20 min instead of 12 min to obtain adequate cooked structure. After baking and cooling, the WWB was sliced into portion sizes and wrapped in aluminum foil, placed in plastic bags and stored in a freezer (– 20 °C). The BKB was placed in a plastic bag at room temperature overnight to obtain a firmer structure and facilitating the slicing into portions. The BKB then followed the procedure similar to the WWB. Before freezing, the crust was removed from the WWB included in the standardized breakfast. The subjects were instructed to take a daily portion of bread from the freezer on the day prior to consumption and to thaw it at ambient temperature, still wrapped in aluminum foil and maintained in the plastic bag. Study design and intervention protocol.

### Study design and intervention protocol

The intervention study was conducted at the Food for Health Science Centre, Lund University, and was completed in May 2016. The study design was crossover, with 3-day dietary intervention periods with BKB and WWB reference, respectively, performed in a randomized order separated with a minimum of a 2-week wash-out period in-between intervention periods. During the wash-out period, the subjects returned to their normal eating habits without diet restrictions or intake of test products. The daily quantity ingested of the test (BKB) and reference (WWB) products during the 3-day study periods was equivalent to 100 g potentially available starch per day (Table 1**)**. The daily amount of product was divided into three portions, and the subjects were instructed to distribute the daily intake of BKB or WWB over the day to suit their diet habits during day 1 and 2. On day 3, half of the daily intake (i.e., 50 g available starch) was distributed equally between meals consumed in the morning and in the afternoon, and the other half (i.e., 50 g available starch) was consumed in the evening at 2100 hours.

The subjects were encouraged to standardize their meal pattern and to avoid alcohol and foods rich in DF during the 3-day intervention periods, and to avoid excessive physical activity on day 3. To facilitate the standardization of meal patterns and to monitor diet intake, the subjects were instructed to provide a meal record during the 3-day intervention period. After intake of the last portion of the BKB or WWB evening meal on day 3, the subjects were fasting until the next morning when the standardized breakfast was provided at the research unit. The subjects arrived at the research unit at 07.30 am, and an intravenous cannula (BD Venflon; Becton Dickinson) was inserted into an antecubital vein for blood sampling. A fasting blood samples were obtained, and subjective appetite variables and breath hydrogen (H_2_) determined, prior to the standardized breakfast provided at approximately 08.00 h. The standardized breakfast corresponded to 50 g available carbohydrates and consisted of 129.6 g WWB, and was ingested together with 2.5 dl tap water. The subjects were instructed to ingest the breakfast within 12 min. During the following experimental time period, the subjects were instructed to maintain low physical activity.

### Collection and analysis of physiological variables

Venous blood samples were collected to analyze the serum (s-) insulin and s-free fatty acids (FFA) and the plasma (p-) glucagon-like peptide-1 (GLP-1), p-GLP-2, p-peptide YY (PYY), p-interleukin-6 (IL-6) and p-c-reactive protein (CRP). Capillary blood samples were taken via finger prick test to determine whole blood glucose concentrations, presenting the results in plasma (HemoCue®B-glucose; HemoCue AB). Indication of colonic fermentation activity was measured as breath hydrogen (H_2_) excretion using a Gastro+ (Bedfont EC60 Gastrolyzer; Bedfont). Measurements of subjective appetite sensations (satiety, hunger and desire to eat) were determined using a 100-mm visual analog scale.

Glucose, H_2_ and appetite sensations were obtained at fasting and at 15, 30, 45, 60, 90, 120, 150 and 180 min after the standardized breakfast. Insulin was analyzed at the same time points, excluding the 180 min time point. GLP-1 was analyzed at fasting and at 30, 60, 90, 120 and 180 min. GLP-2, PYY and IL-6 were analyzed at fasting and at 60, 120 and 180 min. FFA was determined at fasting and at 180 min, and CRP was analyzed at fasting. The plasma tubes were kept on ice (max 30 min) and serum tubes at room temperature (max 1 h room temperature), after blood collection, then centrifuged and immediately frozen after separation and stored in a freezer until analysis (− 40 °C, except samples for GLP-1 which were kept in − 80 °C). Assigned blood-collecting tubes for analysis of p-GLP-1, p-GLP-2 and p-PYY were prepared with a dipeptidyl peptidase-4 (DPPIV) inhibitor (10 µl/ml blood) (Millipore). Tubes prepared with DPPIV inhibitor were kept cold until use, for a maximum of 24 h.

Enzyme immunoassays were used for measuring levels of s-insulin (Mercodia Insulin ELISA, Mercodia AB, Uppsala, Sweden), p-CRP (CRP ELISA Kit, Immundiagnostik AG, Bensheim, Germany), p-IL-6 (Quantikine® HS ELISA, Human IL-6 High Sensitivity HS600B, R&D Systems, Abingdon, UK), p-GLP-1 (GLP-1 (Active 7–36) ELISA 43-GP1HU-E01, ALPCO Diagnostics, Salem, NH), p-GLP-2 (Human GLP-2 EIA YK141, Yanaihara Institute Inc. Shizuoka, Japan) and p-PYY [both PYY (3–36) and PYY (1–36)] (Human PYY EIA YK080, Yanaihara Institute Inc. Shizuoka, Japan). Serum FFA levels were determined using an enzymatic colorimetric method (NEFA C, ACS-ACOD method; Wako Chemicals GmbH).

### Calculations and statistical methods

The statistical evaluations were performed with respect to investigations of effects of treatment (BKB and WWB) and baseline gut microbiota composition (subgroups: HP, LP and HPB), respectively, and interactions thereof, on the test variables included. The effects of treatment and baseline gut microbiota composition, respectively, as well as [Meal * Microbiota] interactions, on physiological markers were analyzed with repeated measures ANOVA (general linear model) applying a 2 × 3 factorial design including two evening meals (BKB and WWB), i.e., ‘Meal’, and three baseline gut microbiota compositions (HP, LP and HPB), i.e., ‘Microbiota’, as independent main variables. The ANOVA was then followed by the post hoc analysis: Tukey’s pairwise multiple comparison method (MINITAB Statistical Software). In addition, a second approach of the factorial design was performed to further elucidate the differences in effects on the physiological markers depending on high- or low abundance of *Prevotella* and *Bacteroides*, excluding the HPB group. Thus, the ‘Microbiota’ variable in the 2 × 2 factorial design included only the two “extreme” groups with respect to ratios of *Prevotella*/*Bacteroides* at baseline, i.e., the HP and LP subgroups. Furthermore, the metabolic responses depending on treatment were also evaluated separately in each microbiota subgroup using one-way ANOVA analysis (MINITAB Statistical Software).

Participants acted as their own control in the statistical evaluation. In the case of unevenly distributed residuals (tested using Anderson–Darling normality test and considered unevenly distributed if *P* < 0.05), transformation using Box Cox was performed on the data prior to the ANOVA analysis. Order of consumption of test and reference product was randomized using the *Random* function in Microsoft Excel 2013 (Washington, USA). In case a value from a subject was missing for the test or reference product, the subject was excluded from that specific calculation. Data are expressed as means ± SEM and values of *P* < 0.05 are considered significant.

The statistical evaluations of glucose and insulin areas were based on both definite values and incremental changes from baseline to postprandial phase at the standardized breakfast consumed at day 4 (the day after the last portion of test product). Calculations of remaining test variables were performed using actual values. The trapezoid model was used to calculate the postprandial area and incremental area under the curve (AUC and iAUC, respectively) for each subject and test product. Graphs and calculations of areas were performed using Graph Prism (version 6; GraphPad Software). Postprandial mean values are reported instead of AUC and iAUC for test variables if the concentrations scarcely changed during the experimental day. Calculations of insulin resistance were made using a homoeostatic model assessment (HOMA-IR) formula [fasting glucose (mmol/l) × fasting insulin (mU/l)/22.5] [20]. Insulin sensitivity was assessed using a modified composite insulin sensitivity (ISI_composite_), also known as Matsuda index, and involved fasting and postprandial measures of b-glucose and s-insulin (ISI_composite_: 10 000/square root of [fasting glucose (mg/dl) × fasting insulin (µU/ml) × mean glucose concentrations 0–120 min (mg min/dl) × mean insulin concentrations 0–120 min (µU min/ml)]) [21]. Modification of Matsuda index refers to that postprandial concentration of b-glucose and s-insulin was obtained after a WWB breakfast consisting of 50 g rapidly available starch instead of 75 g glucose.

## Results

### Baseline abundance of gut bacteria and subject selection

**Fig. 1:**
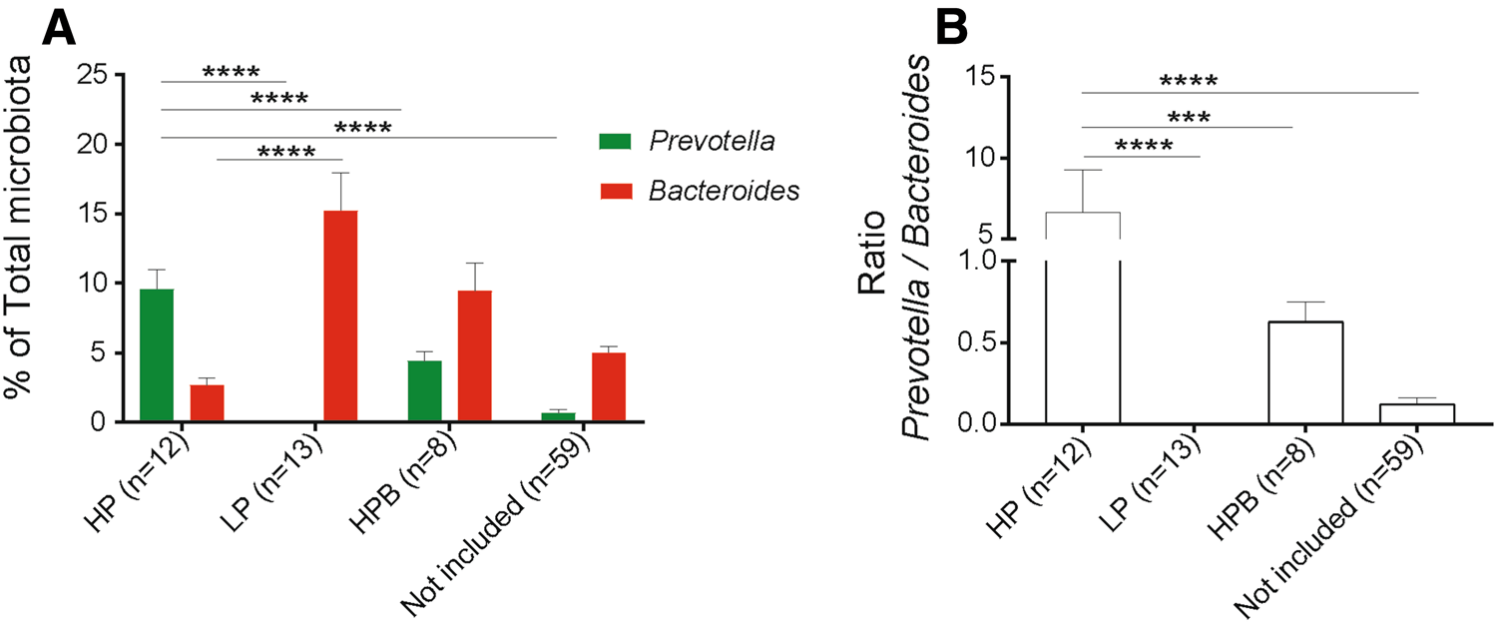
Baseline levels of fecal bacteria and stratification of individuals. **a** Fecal levels of *Prevotella* and *Bacteroides* (determined by quantitative PCR and expressed as fraction of the total microbiota) in participants included and not included in the short-term barley prebiotic intervention. **b** Ratio of *Prevotella* vs *Bacteroides* in participants included and not included in the short-term barley prebiotic intervention. Included HP (High *Prevotella*), LP (Low *Prevotella*) and HPB (High *Prevotella* and *Bacteroides*). ANOVA followed by post hoc analysis—Tukey’s pairwise multiple comparison method was used to compare changes in the levels of *Prevotella* and *Bacteroides* and the ratio *Prevotella*/*Bacteroides* across the four groups. Data are mean ± SEM. ***P < 0.001, ****P < 0.0001

To investigate if the baseline gut microbiota composition, in particular the abundance of *Prevotella* and the ratio *Prevotella*/*Bacteroides*, can identify individuals that will respond to short-term barley prebiotic intervention with improved glucose metabolism, we first quantified the abundance of *Prevotella* and *Bacteroides* in feces of 99 subjects. From this cohort, we identified 15 subjects with highest abundance of *Prevotella* and lowest abundance of *Bacteroides* (group HP). Based on this, we selected 15 subjects with the lowest abundance of *Prevotella* and highest abundance of *Bacteroides* that formed the LP group, and 10 subjects with high abundance of both bacteria (group HPB) to be included in the short-term barley intervention. Of the selected subjects, 12 HP subjects, 13 LP subjects and 8 HPB subjects (Table 2**)** completed the intervention and were included in the statistical evaluation (Fig. 1; Table 3); see the “Materials and methods” section for a comprehensive description regarding selection of individuals and exclusion criteria. The microbiota composition with respect to *Prevotella* and *Bacteroides* of the 59 subjects that were not included in the short-term intervention, are displayed in Fig. 1.

**Table 2:** Age and BMI of the subjects in the three different microbiota subgroups as well as in the total group

Microbiota group	Gender (*n* subjects)	Age (years)	BMI (kg/m^2^*)*
HP (*n* = 12)	3 M/9 W	60.9 ± 7.1	24.1 ± 3.3
LP (*n* = 13)	2 M/11 W	65.8 ± 5.1	24.2 ± 2.7
HPB (*n* = 8)	4 M/4 W	65.8 ± 2.9	23.6 ± 3.9
Total (*n* = 33)	9 M/24 W	64.0 ± 5.8	24.0 ± 3.2

Data including age and BMI are presented as means ± SD

*PPrevotella*,* BBacteroides, M* men, *W* women

**Table 3:** Abundance of *Prevotella* and *Bacteroides*, and *Prevotella*/*Bacteroides* ratio of the subjects in the three different microbiota subgroups

Microbiota group	*Prevotella*, 16S copies	*Bacteroides*, 16S copies	P/B ratio
HP (*n* = 12)	1.32 × 10^8^ ± 1.05 × 10^8^	3.90 × 10^7^ ± 3.29 × 10^7^	6.67 ± 8.93
LP (*n* = 13)	1.12 × 10^5^ ± 1.13 × 10^5^	1.43 × 10^8^ ± 8.77 × 10^7^	0
HPB (*n* = 8)	5.27 × 10^7^ ± 4.56 × 10^7^	1.00 × 10^8^ ± 7.76 × 10^7^	0.63 ± 0.34

Data are presented as means ± SD

*P/B ratio Prevotella*/*Bacteroides* ratio

### Effects of BKB vs WWB interventions on metabolic markers in subgroups varying in baseline gut microbiota composition

To investigate if abundance of *Prevotella* and *Prevotella*/*Bacteroides* ratios can predict the metabolic response to BKB, we analyzed the metabolic responses in the subjects from the different microbiota subgroups (HP, HPB and LP).

#### Glucose and insulin

**Fig. 2:**
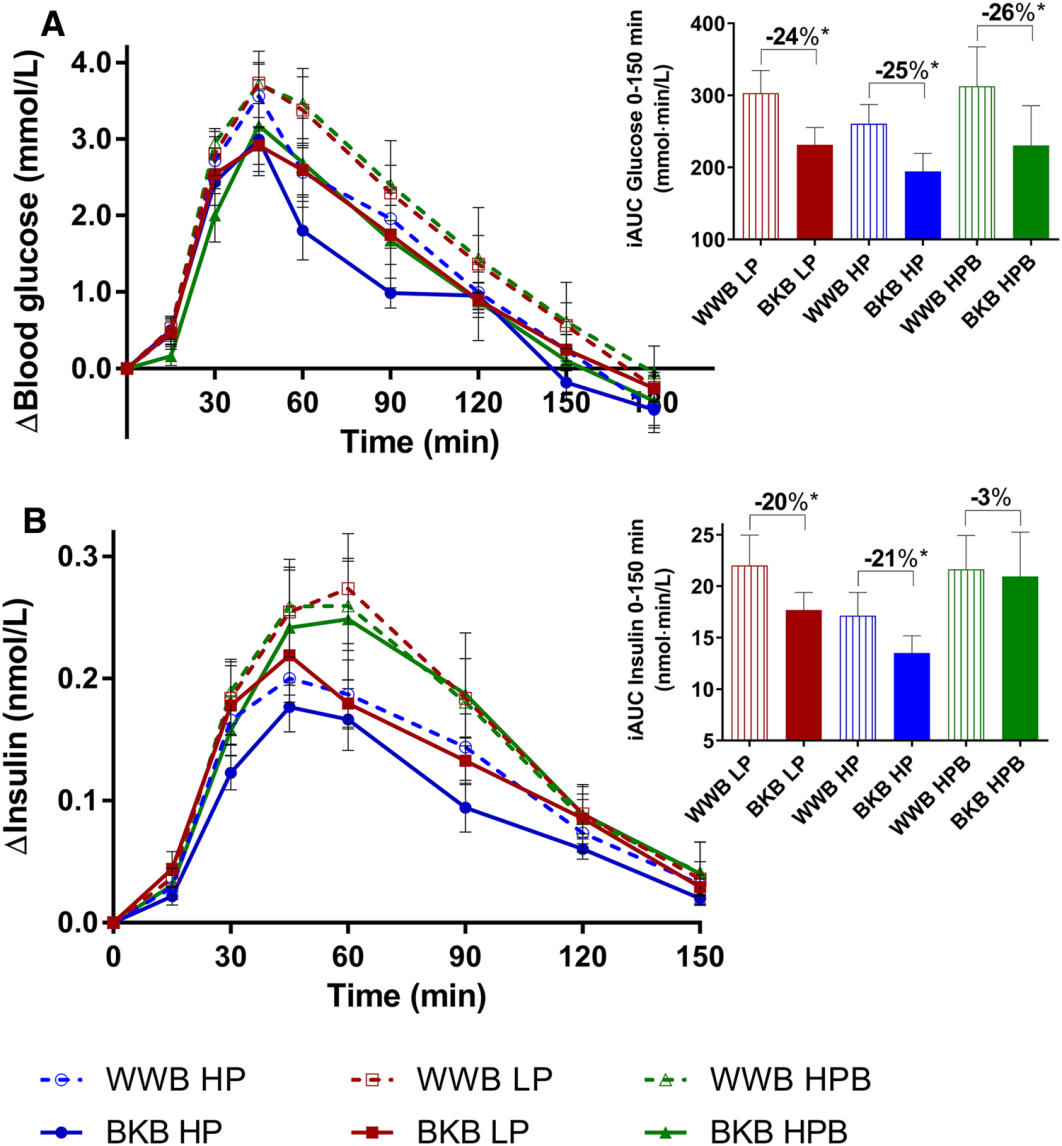
Incremental b-glucose (**a**) and s-insulin (**b**) responses post the standardized breakfast after BKB or WWB interventions, respectively, in subgroups varying in baseline gut microbiota composition. One-way ANOVA was used to compare the metabolic responses depending on intervention in each subgroup. The percentage corresponds to differences in concentration of test variables after BKB intervention compared to WWB intervention. *BKB* barley kernel bread, *WWB* white wheat bread, *HP* high *Prevotella*, *LP* low *Prevotella* and *HPB* high *Prevotella* and *Bacteroides*. Data are mean ± SEM. *P < 0.05

A main effect of treatment was observed on incremental postprandial b-glucose responses (iAUC 0–150 min) to the standardized breakfast (2 × 3 factorial design, *P* < 0.01), indicating that the BKB intervention significantly lowered the b-glucose responses, compared to the WWB, in all three subgroups, i.e., independently of the baseline gut microbiota (Fig. 2). No main effects of intervention were detected on insulin responses to the standardized breakfast. Instead, baseline gut microbiota tended to impact the incremental postprandial s-insulin responses (iAUC 0–150 min) post the standardized breakfast (2 × 3 factorial design, *P* = 0.070), independently of intervention (Fig. 2).

The HPB group was excluded to facilitate the elucidation of differences between the “extremes” with respect to the abundance of *Prevotella* (HP vs LP). When comparing s-insulin responses (iAUC 0–150 min) in these subgroups, we observed that the HP group had an overall lower insulin response, compared to the LP group (2 × 2 factorial design, *P* < 0.05) (Fig. 2). No main effects of treatment or baseline microbiota were observed on fasting b-glucose and s-insulin concentrations (both ANOVA 2 × 3 and 2 × 2 factorial designs, *P* > 0.05) (Table 4).

**Table 4:** Fasting and postprandial measures of b-glucose, s-insulin and insulin sensitivity post the standardized breakfast after BKB or WWB, respectively, in subgroups varying in baseline gut microbiota composition

	WWB		BKB		
	Mean	SEM	Mean	SEM	%Δ^a^
Glucose					
b-Glucose, fasting (mmol/L)					
HP (*n* = 12)	5.6	0.1	5.6	0.1	1
LP (*n* = 13)	5.3	0.2	5.5	0.2	6*
HPB (*n* = 8)	5.5	0.2	5.7	0.2	5
b-Glucose, AUC 0–150 min (mmol·min/L)					
HP (*n* = 12)	1090	38	1030	30	– 6^†^
LP (*n* = 13)	1088	39	1062	39	– 2
HPB (*n* = 8)	1127	67	1077	59	– 4
Insulin					
s-Insulin, fasting (nmol/L)					
HP (*n* = 12)	0.030	0.004	0.032	0.005	8
LP (*n* = 13)	0.029	0.003	0.027	0.003	– 6
HPB (*n* = 8)	0.029	0.005	0.033	0.006	13
s-Insulin, AUC 0–150 min (nmol min/L)					
HP (*n* = 12)	21.6	2.7	18.3	2.2	– 15*
LP (*n* = 13)	26.4	3.3	21.7	2.0	– 18*
HPB (*n* = 8)	26.0	4.0	25.9	5.0	0
Insulin sensitivity					
HOMA-IR					
HP (*n* = 12)	1.23	0.17	1.34	0.22	8
LP (*n* = 13)	1.12	0.14	1.14	0.13	2
HPB (*n* = 8)	1.22	0.26	1.42	0.26	16
ISI_composite_					
HP (*n* = 12)	9.44	1.14	11.05	1.96	17
LP (*n* = 13)	8.96	0.98	9.59	1.06	7
(*n* = 8)	10.2	2.43	8.97	1.68	– 12

Data are presented as mean ± SEM

*BKB* barley kernel bread, *WWB*, white wheat bread

*Different from WWB *P* < 0.05, ^†^*P* = 0.067 (One-way ANOVA)

^a^The percentage change is calculated as the difference from the WWB

When investigating the treatment effects on b-glucose- and s-insulin concentrations in each subgroup separately (using one-way ANOVA), it was observed that 3-day BKB intervention, in comparison with the WWB intervention, increased the fasting b-glucose concentrations in the LP group (*P* < 0.05), whereas no such effects were seen in the other two subgroups (*P* > 0.05) (Table 4). The incremental postprandial s-insulin responses after the standardized breakfast (AUC 0–150 min and iAUC 0–150 min) were decreased after BKB intervention compared with WWB intervention in two subgroups: HP and LP, (*P* < 0.05, Table 4; Fig. 2). In addition, in the HP group, the BKB intervention showed a trend (− 6%, *P* = 0.067) towards lowering the postprandial b-glucose response (AUC 0–150 min), see Table 4 and Fig. 2.

#### Gut hormones

**Fig. 3:**
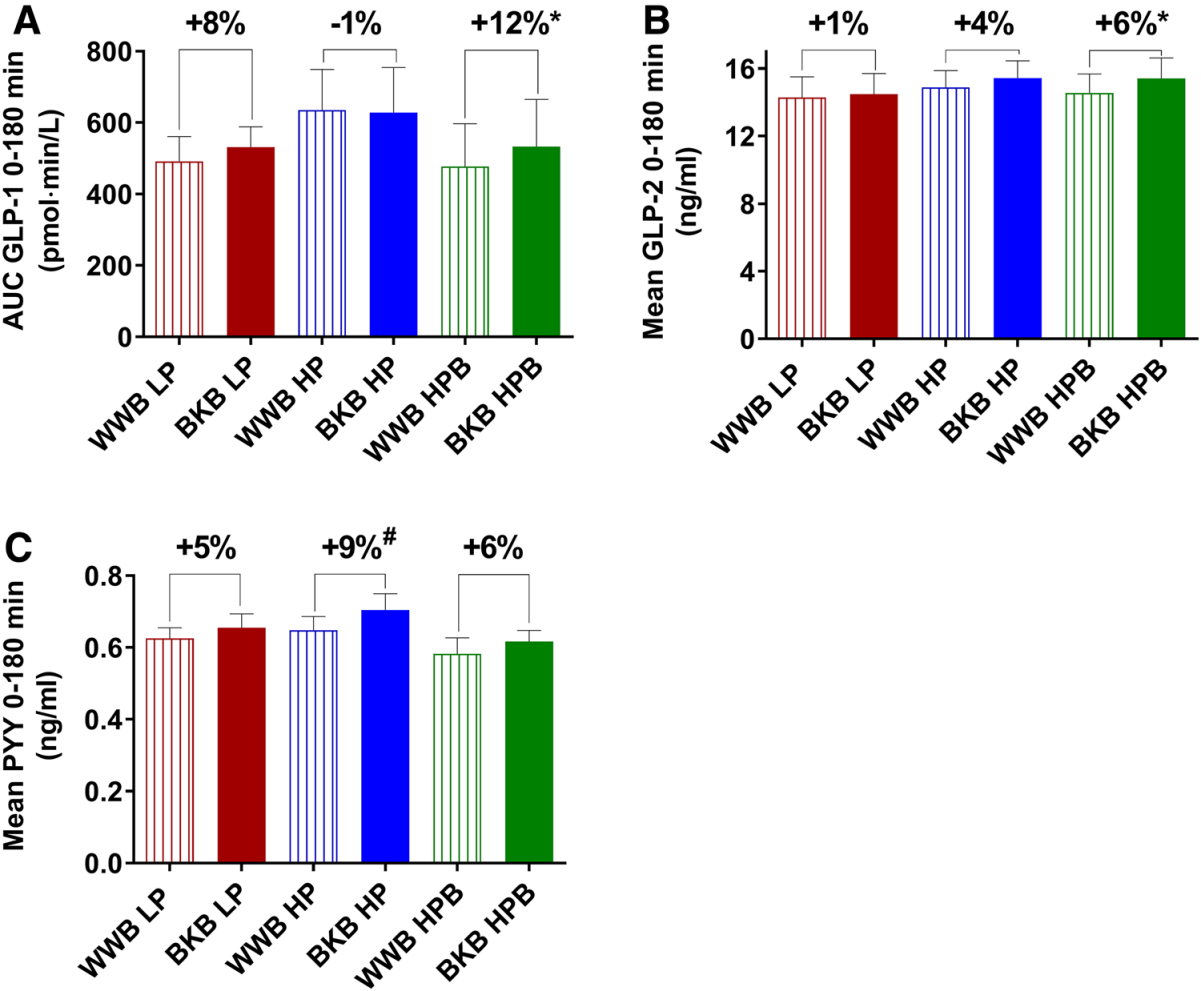
Mean concentrations of p-GLP-1 (**a**), p-GLP-2 (**b**) and p-PYY (**c**) after BKB or WWB intervention, respectively, in subgroups varying in gut microbiota composition. One-way ANOVA was used to compare the metabolic responses depending on intervention in each subgroup. The percentage corresponds to differences in concentration of test variables after BKB intervention compared to WWB intervention. *GLP* glucagon-like peptide, *PYY* peptide YY, *BKB* barley kernel bread, *WWB* white wheat bread, *HP* high *Prevotella, LP* low *Prevotella* and *HPB* high *Prevotella* and *Bacteroides*. Data are mean ± SEM. *P < 0.05, ^#^*P* = 0.06

No significant main effects of treatment or baseline microbiota composition were observed at fasting or with respect to mean concentrations of the gut hormones PYY, GLP-1 or GLP-2 using the ANOVA 2 × 3 (all 3 subgroups) or 2 × 2 (without HPB) factorial designs (Fig. 3 and data are displayed in Online resource 1). However, evaluation of treatment effects on gut hormone concentrations in each subgroup separately (using one-way ANOVA) displayed that the group HPB acquired significantly increased mean GLP-1 and GLP-2 concentrations (0–180 min) with 12 and 6% respectively, after BKB intake compared to after WWB (*P* = 0.018 and *P* = 0.034) (Fig. 3 and Online resource 1). In the HP group, there was a trend towards increased fasting concentration of p-PYY (11%, *P* = 0.050) and mean concentrations of p-PYY (0–180 min, + 9%, *P* = 0.060) in the morning the day after the BKB intervention, compared to after WWB intervention (Fig. 3 and Online resource 1).

#### Breath hydrogen

Breath H_2_ levels at both the fasting and mean (0–180 min) displayed a main effect of treatment (2 × 3 factorial design, *P* < 0.001) and of baseline gut microbiota composition (2 × 3 factorial design, *P* < 0.05) (data are presented in Online resource 1). The post hoc analysis (Tukey’s method) showed that the BKB intervention significantly increased the breath H_2_ excretion in all subgroups, compared to WWB intervention (*P* < 0.001), and revealed that fasting H_2_ levels were higher in the LP group compared with the HP group (*P* = 0.017) (Online resource 1).

#### Markers of inflammation

**Fig. 4:**
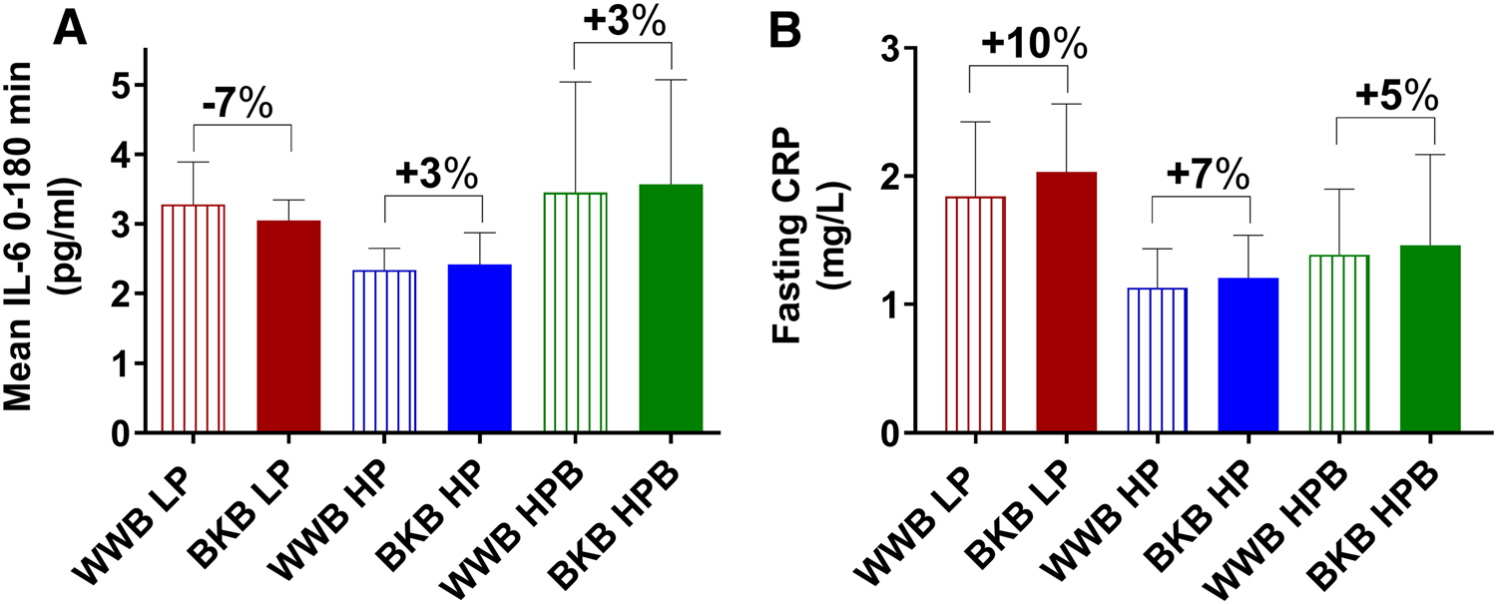
Plasma concentrations of IL-6 (**a**) after the standardized breakfast and fasting values of CRP (**b**) after BKB or WWB intervention, respectively, in subgroups varying in gut microbiota composition. One-way ANOVA was used to compare the metabolic responses depending on intervention in each subgroup. The percentage corresponds to differences in concentration of test variables after BKB intervention compared to WWB intervention. *IL* interleukin, *CRP* c-reactive protein, *BKB* barley kernel bread, *WWB* white wheat bread, *HP* high *Prevotella, LP* low *Prevotella* and HPB high *Prevotella* and *Bacteroides*. Data are mean ± SEM

No significant main effects depending on treatment or baseline gut microbiota composition were observed regarding the concentrations of p-IL-6 and p-CRP in the ANOVA 2 × 3 factorial design (Fig. 4 and data are displayed in Online resource 2). However, comparing results in the HP and LP subgroups only (2 × 2 factorial design), a main effect of baseline gut microbiota composition was detected on concentrations of p-IL-6 (mean 0-180 min, *P* = 0.045), revealing lower IL-6 concentrations in the HP group (Fig. 4 and Online resource 2). In addition, the comparison of the CRP fasting concentrations in the subgroups HP and LP displayed a trend towards a main effect of baseline gut microbiota composition (2 × 2 factorial design, *P* = 0.084), indicating a trend towards lower CRP concentrations in the HP group (Fig. 4 and Online resource 2). No significant differences in concentrations of p-IL-6 or p-CRP were seen within the separate microbiota subgroups depending on treatment (one-way ANOVA, *P* > 0.05) (Fig. 4 and Online resource 2).

#### Free fatty acids

No significant main effects depending on treatment or baseline gut microbiota composition were observed regarding the concentrations of s-FFA in the ANOVA 2 × 3 or 2 × 2 factorial design (data are displayed in Online resource 2). Neither were there any significant differences in concentrations of FFA within the separate microbiota subgroups depending on treatment (one-way ANOVA, *P* > 0.05).

#### Subjective appetite ratings

**Fig. 5:**
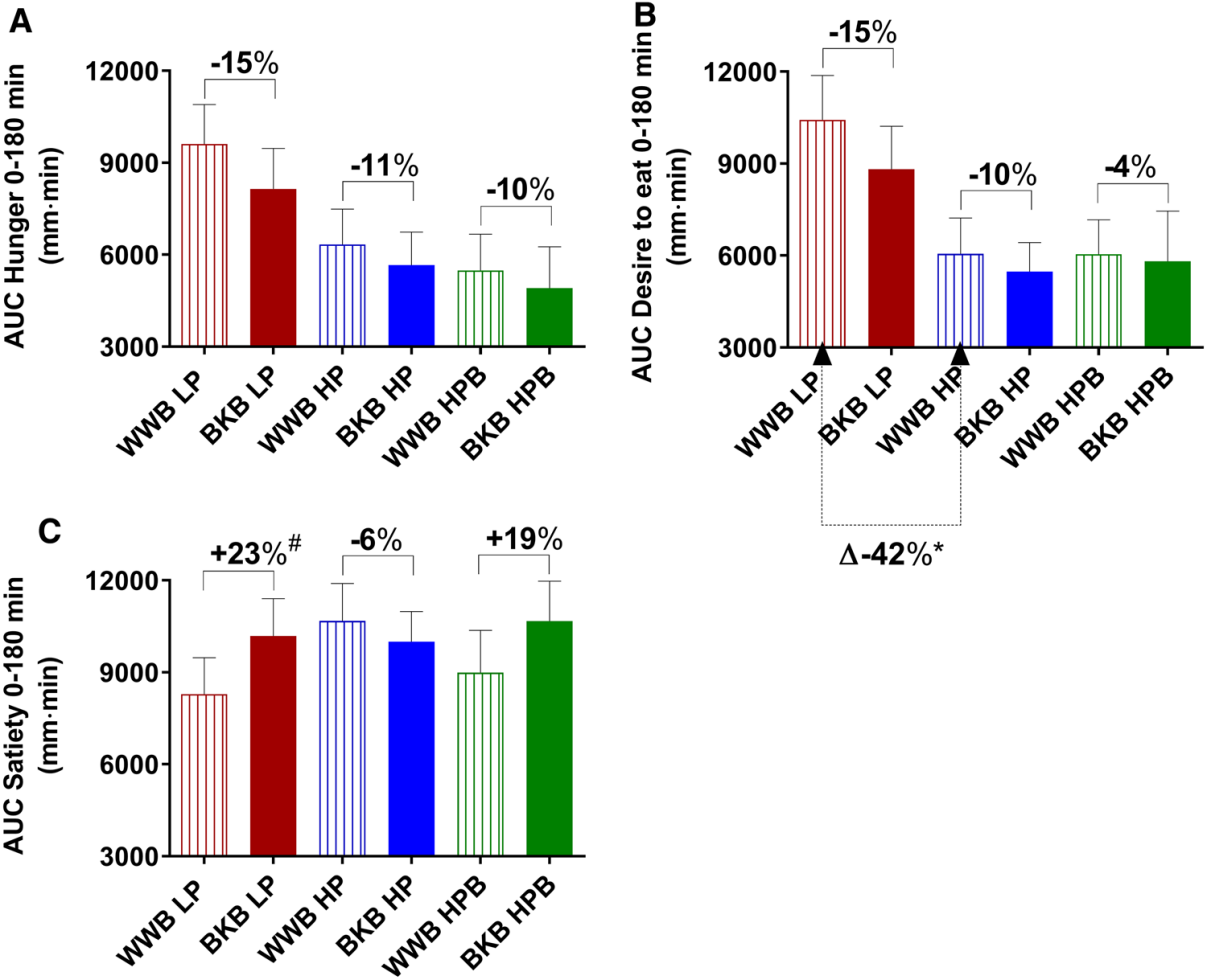
Subjective appetite ratings [subjective hunger (**a**), desire to eat (**b**) and satiety (**c**)] post standardized breakfast after BKB or WWB intervention, respectively, in subgroups varying in gut microbiota composition. One-way ANOVA was used to compare the metabolic responses depending on intervention in each subgroup. The percentage corresponds to differences in concentration of test variables after BKB intervention compared to WWB intervention. *BKB* barley kernel bread, *WWB* white wheat bread, *HP* high *Prevotella, LP* low *Prevotella* and *HPB* high *Prevotella* and *Bacteroides*. Data are mean ± SEM. ^#^P = 0.065

The postprandial appetite ratings ‘hunger’ and ‘desire to eat’ (AUC 0–180 min) displayed main effects of baseline gut microbiota composition (2 × 3 factorial, hunger, *P* < 0.05; desire to eat, *P* < 0.01) (Fig. 5 and data are presented in Online resource 3). The post hoc analysis (Tukey’s method) showed a significantly higher sensations of hunger and desire to eat in the LP group compared to both the HP group (hunger, *P* < 0.05; desire to eat, *P* < 0.01) and the HPB group (both variables, *P* < 0.05) (Fig. 5 and Online resource 3). No significant main effects were seen on subjective appetite ratings depending on treatment. However, investigations of effects of treatment in each subgroup separately, analyzed using one-way ANOVA, revealed significant effects of treatment on appetite ratings in the LP group. Consequently, after the BKB intervention, the subgroup LP significantly decreased sensations of hunger at fasting (− 28%, *P* < 0.05) and increased fasting satiety (+ 92%, *P* < 0.01) (Online resource 3), and showed a trend to decrease postprandial desire to eat (AUC 0–180 min, − 15%, *P* = 0.082) (Fig. 5 and Online resource 3), compared to the ratings after WWB intake.

## Discussion

The purpose of this study was to investigate if the abundance of gut *Prevotella* and the ratio of *Prevotella*/*Bacteroides* at baseline may predict the effects of barley kernel-based food on cardiometabolic risk variables in a time perspective of, e.g., 11–13.5 h after intake. Therefore, subjects were stratified into different subgroups (HP, LP and HPB) depending on the gut microbiota composition. We evaluated both the effects of the short-term 3-day interventions with BKB vs WWB and effects of microbiota subgroups on cardiometabolic test variables. Effects of the interventions were also evaluated within each microbiota subgroup. The current design, 3-day long intervention with BKB, was based on previous findings where 3 days of intake of BKB was sufficient to observe acute effects of gut microbiota and glucose metabolism [10].

All subgroups responded to the BKB intervention with improved glucose tolerance 11 h after intake as compared with the WWB intervention, thus, the results were not dependent on the subgroup stratification. Instead, we identified significant differences in cardiometabolic test markers between the microbiota subgroups independently of the intervention. Furthermore, the beneficial response to BKB can be driven by a substrate depending expansion of *Prevotella*, regardless of the baseline levels gut *Prevotella*, which can mediate the improved glucose metabolism, in agreement to our previous findings [10].

Colonic fermentation of DF endogenous to barley kernels has been related to improved cardiometabolic risk markers in healthy subjects [6, 7]. However, high variability between subjects in the metabolic responses to food, e.g., postprandial glycemic responses, or SCFA production (in particular butyrate), has previously been observed, which may be related to gut microbiota composition [11, 12, 22]. In this context, we have previously shown that improved glucose tolerance after a short-term intervention with BKB was related to an enrichment of gut *Prevotella*, where *P. copri* was the most prevalent [10]. In addition, subjects responding to BKB with an improved glucose tolerance tended to have an elevated abundance of *Prevotella* also at baseline, prior to the BKB intervention. *Prevotella* can ferment a wide spectrum of complex carbohydrates, such as arabinoxylans and β-glucans, i.e., DF abundant in barley [23]. Populations living in rural areas ingesting diets rich in DF have higher abundance of *Prevotella* compared to those on a Western diet [24]. Accordingly, *Prevotella* has been associated with high intake of carbohydrates/dietary fiber [25].

With respect to the metabolic effects of barley kernel-based products, the results in the current study were in accordance with previous results showing beneficial effects on markers of glucose regulation, gut hormones, subjective appetite variables, and increased breath H_2_ excretion [6, 7, 26, 27]. Consequently, compared to WWB, the BKB intervention resulted in improvements with respect to glucose tolerance in all subgroups, and insulin responses decreased in the subgroups HP and LP. Furthermore, gut hormones (GLP-1 and GLP-2) increased after BKB in the HPB group in the postprandial phase, whereas there was a trend (*P* = 0.05) towards increased PYY fasting concentrations in the HP group. In addition, the BKB intervention resulted in decreased sensation of hunger and increased satiety after BKB intervention in the LP group. Breath H_2_ excretion was increased in all subgroups after the BKB intervention.

Interestingly, the main effects of baseline gut microbiota composition (HP, LP or HPB) were on appetite variables (hunger and desire to eat) and breath H_2,_ independent of the intervention. Thus, although the LP group improved appetite ratings after the BKB intervention, the post hoc analysis revealed that both subgroups with higher gut *Prevotella* abundance, i.e., the subgroups HP and HPB, in general displayed improved appetite sensations compared with the LP group with respect to less hunger and less desire to eat, independently of intervention. In addition, it was shown that the HP group had significantly lower breath H_2_ concentrations at fasting, compared to the LP group. These findings further suggest differences in the gut microbiota fermentative activities between these two groups. Furthermore, the differences in insulin responses to the standardized breakfast between the microbiota subgroups that differ the most in composition, i.e., the HP and LP groups, revealed a significant effect of baseline gut microbiota composition, demonstrating that a high abundance of *Prevotella* and high *Prevotella*/*Bacteroides* ratio at baseline may be advantageous with respect to postprandial glucose homeostasis, compared to a low baseline *Prevotella* and low *Prevotella*/*Bacteroides* ratio.

Upon designing the study, we included 99 subjects with the intention to identify 20 subjects to be included in the HP group and 20 subjects in the LP group. However, following the initial microbial screening, we could not identify 20 subjects with high or low *Prevotella*, which limits the study design. Surprisingly, we identified subjects with relatively high levels of both *Prevotella* and *Bacteroides* (the HPB group), which is interesting as taxa belonging to these genera usually are believed to compete for the same niche [28].

In the present study, we focused on the potential changes in cardiometabolic test variables after BKB compared to WWB in healthy subjects after a short-term intervention depending on specific bacteria ratios. For future work, it would be interesting to design a longer term study and it could also be of interest to focus on prediabetic cohort and/or different ages. Furthermore, the screening criteria for healthy subjects would also benefit from including HbA1c measurements to obtain further indication of the glycemic status.

In summary, the BKB intervention resulted in improved glucose tolerance after the forthcoming standardized breakfast in all microbiota subgroups, suggesting overall prebiotic and anti-diabetic potentials of barley DF independently of *Prevotella*/*Bacteroides* ratio at baseline. There were no differences between the microbiota subgroups in response to BKB, in comparison to WWB, with respect to the tested variables. Thus, our results do not support the hypothesis that the *Prevotella*/*Bacteroides* ratio at baseline can be used to stratify metabolic responders and non-responders to the prebiotic mixture present in BKB. However, these findings do not exclude that the beneficial effects of DF are mediated by *Prevotella*-dependent fermentation. In addition, based on reduced insulin responses, reduced inflammatory markers and improved subjective appetite ratings in the HP group compared to the LP group (independent of intervention), the results of the current study suggest that higher *Prevotella*/*Bacteroides* ratio may be advantageous with respect to cardiometabolic regulation, and may play a preventive role in the development of obesity, T2D and CVD.

## Electronic supplementary material

Below is the link to the electronic supplementary material.

Supplementary material 1 (PDF 114 KB)

Supplementary material 2 (PDF 78 KB)

Supplementary material 3 (PDF 94 KB)
