# Supplementary material for: Abundance of gut Prevotella at baseline and metabolic response to barley prebiotics
Source: Eur J Nutr. 2018 Jul 25;58(6):2365–76. doi: 10.1007/s00394-018-1788-9 (PMC6689319; doi:10.1007/s00394-018-1788-9)
Supplement: Supplementary file 1 — Supplementary material 1 (PDF 114 KB) [file 394_2018_1788_MOESM1_ESM.pdf]

Online resource 1 Fasting and postprandial measures of gut hormones and breath hydrogen responses in the microbiota subgroups in the morning following the interventions with BKB or WWB intake

	WWB		BKB		
	Mean	SEM	Mean	SEM	%Δ[1]
p-PYY, fasting (ng/mL,)					
HP, n=12	0.62	0.04	0.69	0.04	***11†***
LP, n=13	0.60	0.03	0.64	0.04	*7*
HPB, n=8	0.59	0.04	0.61	0.03	*3*
p-PYY, mean 0-180 min (ng/mL)					
HP, n=12	0.65	0.04	0.70	0.05	***9‡***
LP, n=13	0.63	0.03	0.66	0.04	*5*
HPB, n=8	0.58	0.04	0.62	0.03	*6*
p-GLP-2, fasting (ng/mL)					
HP, n=12	14.1	1.1	14.4	0.9	*2*
LP, n=13	13.4	1.2	13.5	1.1	*0*
HPB, n=8	14.1	1.2	14.3	1.3	*1*

p-GLP-2, mean 0-180 min (ng/mL)					
HP, n=12	14.9	1.0	15.4	1.0	*4*
LP, n=13	14.3	1.2	14.5	1.2	*1*
HPB, n=8	14.6	1.1	15.4	1.2	***6****
p-GLP-1, fasting (pmol/L)					
HP, n=12	3.06	0.64	3.09	0.68	*1*
LP, n=12	2.39	0.35	2.27	0.33	*-5*
HPB, n=8	2.32	0.82	2.35	0.81	*1*
p-GLP-1, AUC 0-180 min (pmol·min/L)					
HP, n=12	635.0	114.0	628.0	126.0	*-1*
LP, n=12	491.5	69.2	531.7	56.9	*8*
HPB, n=8	477.0	120.0	533.0	132.0	***12****
breath H_2, fasting (ppm)					
HP, n=12	15.4	3.4	34.5	7.5	***124*****
LP, n=13	30.8	6.6	67.1	16.5	***118****
HPB, n=8	25.0	8.0	61.0	11.9	***144****
breath H_2, weighted mean 0-180 min (ppm)					

HP, n=12	9.6	1.7	40.4	11.0	***322***
LP, n=13	17.2	4.2	57.8	12.0	***235***
HPB, n=8	16.3	3.9	54.6	11.0	***234***

[1] The percentage change is calculated as the difference from the WWB

BKB, barley kernel bread; WWB, white wheat bread; PYY, peptide YY; GLP, glucagon-like peptide; H_2, hydrogen

*Different from WWB P < 0.05, **P < 0.01, *** P < 0.001, †P = 0.050, ‡P = 0.060 (One-way ANOVA)

Data are presented as mean ± SEM
