# Supplementary material for: Abundance of gut Prevotella at baseline and metabolic response to barley prebiotics
Source: Eur J Nutr. 2018 Jul 25;58(6):2365–76. doi: 10.1007/s00394-018-1788-9 (PMC6689319; doi:10.1007/s00394-018-1788-9)
Supplement: Supplementary file 2 — Supplementary material 2 (PDF 78 KB) [file 394_2018_1788_MOESM2_ESM.pdf]

Online resource 2 S-FFA and inflammatory marker responses in the microbiota subgroups post the standardized breakfast, following BKB- or WWB intervention

	WWB		BKB		
	Mean	SEM	Mean	SEM	%Δ[1]
s-FFA, fasting (mmol/L)					
HP, n=12	0.49	0.06	0.48	0.06	*-3*
LP, n=13	0.45	0.04	0.51	0.05	*14*
HPB, n=8	0.48	0.10	0.47	0.06	*-3*
s-FFA, mean 0+180 min (mmol/L)					
HP, n=12	0.36	0.04	0.38	0.06	*5*
LP, n=13	0.38	0.03	0.41	0.04	*6*
HPB, n=8	0.37	0.05	0.37	0.03	*-2*
p-IL-6, fasting (pg/mL)					
HP, n=11	1.68	0.28	1.65	0.24	*-1*
LP, n=12	2.09	0.36	1.95	0.17	*-6*
HPB, n=8	2.93	1.36	2.94	1.31	*0*
p-IL-6, mean 0-180 min (pg/mL)					

HP, n=11	2.34	0.31	2.41	0.46	*3*
LP, n=12	3.28	0.61	3.05	0.30	*-7*
HPB, n=8	3.45	1.59	3.57	1.50	*3*
p-CRP, fasting (mg/L)					
HP, n=11	1.13	0.30	1.21	0.33	*7*
LP, n=12	1.84	0.58	2.03	0.53	*10*
HPB, n=8	1.39	0.51	1.46	0.71	*5*

[1] The percentage change is calculated as the difference from the WWB

BKB, barley kernel bread; WWB, white wheat bread; FFA, free fatty acids; IL, interleukin; CRP, c-reactive protein

Data are presented as mean ± SEM
