# Supplementary material for: Abundance of gut Prevotella at baseline and metabolic response to barley prebiotics
Source: Eur J Nutr. 2018 Jul 25;58(6):2365–76. doi: 10.1007/s00394-018-1788-9 (PMC6689319; doi:10.1007/s00394-018-1788-9)
Supplement: Supplementary file 3 — Supplementary material 3 (PDF 94 KB) [file 394_2018_1788_MOESM3_ESM.pdf]

Online resource 3 Subjective appetite responses in the microbiota subgroups after the standardized breakfast following the intervention with BKB or WWB intake

	WWB		BKB		
	Mean	SEM	Mean	SEM	%Δ[1]
Hunger, fasting (mm)					
HP, n=12	38.0	9.3	44.5	7.7	*17*
LP, n=12	56.4	6.2	40.5	9.0	***-28****
HPB, n=8	36.6	8.8	37.4	8.4	*2*
Hunger, AUC 0-180min (mm·min)					
HP, n=12	6335	1155	5656	1083	*-11*
LP, n=12	9613	1288	8151	1320	*-15*
HPB, n=8	5484	1184	4911	1346	*-10*
Desire to eat, fasting (mm)					
HP, n=12	59.4	7.2	53.0	8.6	*-11*
LP, n=12	64.4	6.2	53.0	8.6	*-18*
HPB, n=8	48.5	10.0	44.6	9.8	*-8*
Desire to eat, AUC 0-180min (mm·min)					

HP, n=12	6058	1170	5476	944	*-10*
LP, n=12	10430	1448	8815	1404	***-15***
HPB, n=8	6044	1125	5808	1646	*-4*
Satiety, fasting (mm)					
HP, n=12	32.6	10.1	39.2	7.8	*20*
LP, n=12	28.8	5.3	55.3	8.7	***92***
HPB, n=8	30.3	7.7	35.8	10.0	*18*
Satiety, AUC 0-180min (mm·min)					
HP, n=12	10674	1221	9999	982	*-6*
LP, n=12	8287	1188	10184	1218	*23*†
HPB, n=8	8988	1384	10666	1304	*19*

[1] The percentage change is calculated as the difference from the WWB

BKB, barley kernel bread; WWB, white wheat bread

* Different from WWB P < 0.05, **P < 0.01, †P = 0.065 (One-way ANOVA)

Data are presented as mean ± SEM
